# Supplementary material for: Changes in parenting behavior in the time of COVID—19: A mixed method approach
Source: PLoS One. 2024 Apr 19;19(4):e0302125. doi: 10.1371/journal.pone.0302125 (PMC11029621; doi:10.1371/journal.pone.0302125)
Supplement: S1 Appendix — (DOCX) [file pone.0302125.s001.docx]

**Appendix S1. Descriptive statistics of the index’s items**

| **Index variables** | **Items** | **Mean** | **SD** | **Median** | **Min** | **Max** |
| --- | --- | --- | --- | --- | --- | --- |
| Perception of the pandemic impact (PI) | 1. The Coronavirus (COVID-19) has impacted me negatively from a financial point of view. (F) | 3.12 | 2.19 | 3 | 1 | 7 |
|  | 2. I have had a hard time getting the resources I needed (food, toilet paper) due to the Coronavirus (COVID-19). (R) | 2.73 | 2.02 | 2 | 1 | 7 |
|  | 3. The Coronavirus (COVID-19) outbreak has impacted my psychological health negatively. (P) | 3.25 | 2.05 | 3 | 1 | 7 |
|  | 4. I have lost job-related income due to the Coronavirus (COVID-19). (F) | 2.47 | 2.22 | 1 | 1 | 7 |
|  | 5. It has been difficult for me to get the things I need due to the Coronavirus (COVID-19). (R) | 2.44 | 1.86 | 2 | 1 | 7 |
|  | 6. I have become depressed because of the Coronavirus (COVID-19). (P) | 2.14 | 1.66 | 1 | 1 | 7 |
| Parenting Behavior (PB) | 1. The COVID pandemic had a NEGATIVE impact on my ability to be a good parent; 1. The COVID pandemic had a POSITIVE impact on my ability to be a good parent  *(Bonding -effective parent)* | 3.51 | 1.31 | 4 | 1 | 5 |
|  | 2. The COVID pandemic had a NEGATIVE impact on my way of giving CHILD AFFECTION; 2. The COVID pandemic had a POSITIVE impact on my way of giving CHILD AFFECTION. *(Affection)* | 3.98 | 1.18 | 4 | 1 | 5 |
|  | 3. The COVID pandemic had a NEGATIVE impact on my way of COMMUNICATING with children; 3. The COVID pandemic had a POSITIVE impact on my way of COMMUNICATING with children *(Communication)* | 3.84 | 1.22 | 4 | 1 | 5 |
|  | 4. The COVID pandemic had a NEGATIVE impact on my way of caring for children; The COVID pandemic had a POSITIVE impact on my way of caring for children *(Caring)* | 3.89 | 1.15 | 4 | 1 | 5 |
|  | 5. The COVID pandemic had a NEGATIVE impact on my way of monitoring and controlling children's activity; 5. The COVID pandemic had a POSITIVE impact on my way of monitoring and controlling children's activity;  *(Monitoring and control)* | 3.73 | 1.31 | 4 | 1 | 5 |
|  | 6. The COVID pandemic had a NEGATIVE impact on my way of resolving conflicts with children; The COVID pandemic had a POSITIVE impact on my way of resolving conflicts with children. *(Conflict)* | 3.46 | 1.15 | 3 | 1 | 5 |
|  | q7. The COVID pandemic had a NEGATIVE impact on my way of GETTING INVOLVED in fun activities with children; 7 The COVID pandemic had a POSITIVE impact on my way of GETTING INVOLVED in fun activities with children. *(Involvement)* | 3.75 | 1.35 | 4 | 1 | 5 |
|  | 8. The COVID pandemic had a NEGATIVE impact on my way of listening to and understanding the NEEDS of my children; 8. The COVID pandemic had a POSITIVE impact on my way of listening to and understanding the NEEDS of my children (Closeness ) | 3.95 | 1.11 | 4 | 1 | 5 |
| Discipline Practices (DP) | 1. You ignore your child when he/she is misbehaving. | 2.66 | .88 | 3 | 1 | 5 |
|  | 2. You take away privileges or money from your child as punishment. | 3.11 | .87 | 3 | 1 | 5 |
|  | 3. You send your child to his/her room as punishment. | 2.85 | .93 | 3 | 1 | 5 |
|  | 4. You yell or scream at your child when he/ she has done something wrong. | 2.89 | 1 | 3 | 1 | 5 |
|  | *5. You calmly explain to your child why his/ her behavior was wrong when he/she misbehaves.* | 3.46 | .89 | 3 | 1 | 5 |
|  | 6.You use time out (make him/her sit or  Stand in corner) as a punishment. | 2.40 | 1.15 | 3 | 1 | 5 |
|  | 7. You give your child extra chores as a punishment. | 2.81 | 1.15 | 3 | 1 | 5 |
| Parent Involvement in School (PIS) | 1. How often does the mother talk with their children about homework? | 3.25 | .87 | 3 | 1 | 5 |
|  | 2. How often does the mother ask their children about what they did at school? | 3.39 | .68 | 3 | 1 | 5 |
|  | 3. How often does the mother go over graded papers with their children? | 3.42 | .73 | 3 | 1 | 5 |
|  | 4. How often does the mother talk with their children about their schoolwork? | 3.47 | .71 | 3 | 1 | 5 |
|  | 5. How often does the mother talk with their children about how they behave at school? | 3.44 | .73 | 3 | 1 | 5 |
|  | 6. How often does the mother talk with their children about doing their best at school? | 3.51 | .72 | 3 | 1 | 5 |
|  | 7. How often does the mother ask their children about one of their teachers at school? | 3.41 | .78 | 3 | 1 | 5 |
