# Supplementary material for: Changes in parenting behavior in the time of COVID—19: A mixed method approach
Source: PLoS One. 2024 Apr 19;19(4):e0302125. doi: 10.1371/journal.pone.0302125 (PMC11029621; doi:10.1371/journal.pone.0302125)
Supplement: S2 Appendix — (DOC) [file pone.0302125.s002.doc]

**Appendix S2. Sociodemographic characteristic of qualitative survey respondents (N=24)**

| **Code 1** | **Age** | **Children attending school** (yes/no) | **Family structure**  (Single mother/  Nuclear family/  Extended family or other combinations | **Residence environment**  (urban/rural) | Bachelor degree (yes/no) | **Number of children**  (one child/more children) | **Income**  (Under 2000 Ron (low)  2000-4000 Ron (medium)  Over 4000 Ron (high)  n.r.) | Employment situation  (Employee/  Unemployed/  Housewife) | Way of working (Exclusively at home  Hybrid (at home and at work)  Exclusively at work) |
| --- | --- | --- | --- | --- | --- | --- | --- | --- | --- |
| 1 | 49 | yes | Nuclear family | Urban | yes | 1 | - | Housewife | - |
| 2 | 44 | yes | Extended family | Urban | yes | 1 | medium | Employee | Hybrid |
| 3 | 39 | yes | Nuclear family | Urban | yes | 1 | high | Employee | Hybrid |
| 4 | 38 | yes | Nuclear family | Urban | yes | 3 | medium | Employee | Didn't work in pandemic period |
| 5 | 39 | yes | other combinations | Urban | yes | 2 | high | Employee | Hybrid |
| 6 | 30 | yes | Nuclear family | Urban | yes | 2 | high | Employee | Exclusively at work |
| 7 | 38 | no | Nuclear family | Urban | yes | 1 | high | Employee | Hybrid |
| 8 | 39 | yes | Nuclear family | Urban | yes | 2 | high | Employee | Hybrid |
| 9 | 38 | yes | Nuclear family | Rural | yes | 2 | medium | Employee | Exclusively at home |
| 10 | 34 | no | Nuclear family | Rural | yes | 1 | high | Employee | Exclusively at work |
| 11 | 39 | yes | Nuclear family | Urban | yes | 2 | high | Employee | Hybrid |
| 12 | 40 | yes | Nuclear family | Urban | yes | 2 | high | Unemployed | - |
| 13 | 41 | yes | Nuclear family | Urban | yes | 2 | medium | Employee | Exclusively at home |
| 14 | 36 | yes | Nuclear family | Urban | no | 3 | low | Not specified | Not specified |
| 15 | 33 | no | Nuclear family | Urban | yes | 2 | medium | Unemployed | - |
| 16 | 44 | no | other combinations | Not specified | no | 2 | low | Employee | Exclusively at work |
| 17 | 30 | no | Nuclear family | Urban | yes | 1 | medium | Not specified | Not specified |
| 18 | 41 | yes | Nuclear family | Urban | yes | 1 | medium | Employee | Hybrid |
| 19 | 30 | no | Nuclear family | Urban | yes | 1 | high | Employee | Hybrid |
| 20 | 46 | yes | Nuclear family | Urban | yes | 1 | high | Employee | Exclusively at home |
| 21 | 36 | no | Nuclear family | Rural | yes | 1 | high | Employee | Hybrid |
| 22 | 44 | yes | Nuclear family | Urban | yes | 1 | low | Health retirement | - |
| 23 | 34 | yes | other combinations | Urban | yes | 2 | medium | Unemployed | - |
| 24 | 39 | no | Nuclear family | Urban | yes | 1 | high | Employee | Exclusively at work |
