# Supplementary material for: Changes in parenting behavior in the time of COVID—19: A mixed method approach
Source: PLoS One. 2024 Apr 19;19(4):e0302125. doi: 10.1371/journal.pone.0302125 (PMC11029621; doi:10.1371/journal.pone.0302125)
Supplement: S3 Appendix — (DOCX) [file pone.0302125.s003.docx]

**Appendix S3. Results of the qualitative thematic analysis**

| ***Thematic categories*** | ***Themes*** | ***Representative quotations*** |
| --- | --- | --- |
| Effective parenting (bonding) | Perceived positive changes | ” I learned to be more patient, to have more realistic expectations and to maintain the limits I set. For example, I learned to accept that a 5 year old child cannot play by himself more than 60 minutes”.  Connection: I think that this year we felt connected more than never, and my husband and I dedicated more time to being present and discussing with our children” |
|  | Perceived negative changes | “I consider that this period deteriorated the relation if we take into account the peace and psychological balance on both the parents and the children, but also the mother’s ability to remain calm.” “the work volume of parents, the lack of quality time spent with the family, stress and the daily “run” after anything else rather than what was important for the child, led to the deterioration of the relationship, to lack of playing with the children, to the “lies” of the children (playing on the computer, or homework), and to a decrease in trusting the parent/the child” |
| Affection | Perceived positive changes | “Yes, there were many moments when seeing their emotions and how they felt, I wanted to hug them, kiss them and tell them that I loved them. I simply saw them more, I did not estrange myself from the family the way I do when I go to the office. I would say that we hugged more. I strongly feared, especially in the beginning, that something could happen to me or my partner and they would remain alone, without support.”  “Yes, I felt the need to manifest my affection more often, in order to compensate for the mistakes I made. I bought more toys for them, I cut my resting hours at night, just to be able to connect and talk at least once a day before going to bed. I kissed them and pampered them whenever I had the occasion.” |
|  | Perceived negative changes | “I became overprotective, I did not let them go outside or to get into contact with anybody”  “In this period, I cared more for my child because of the fear of the disease. Maybe I overreacted and I became possessive because we all contracted the virus”  “They have all my moral support. I understand that they were affected just as we were. I try to be there for them: to play on the carpet when they need to, to take sides in turn, to compensate for what they miss, even though I not 100% authentic.” |
| Communication | Perceived positive changes | “We spent more time together during the emergency period, we talked about the current situation, to better understand what is happening, what measures should be taken”  “We talked more about what he feels, what he misses, what he liked and didn't like last year, about the present and our plans for the future. " |
|  | Perceived negative changes | “It is difficult for me when they are resilient: for example my little daughter, who sometimes gets very angry and does not let me calm her down, or who on some days feels nothing is good from morning to evening and nothing seems to help her. And sometimes I lose my temper, I get angry and shout or tease her but someone jumps to my aid and I recover quickly and we hug. We apologize, we calm down and we resume the talks and most of the time we realized that the children are fighting with great fears and they have strong feelings that have nothing to do with the episode from which the scandal started. I am small and humble and I am sorry that I did not have the patience and calm to support her from the beginning and I try to remember this until next time. ”  “A lot of the time, as a parent, I ended up raising my voice and arguing because he wasn't doing his schoolwork the way he used to. I never "screamed" at him as I did during this time. |
| Carrying | Perceived positive changes | “Conveying the meaning of responsibilities for the good of the family. Each of us can do something to make each day more beautiful”,  *“*Cooperation, children were also involved in the housework, we worked more together "  “ We learned to trust and support each other” |
|  | Perceived negative changes | I don't think this period has changed the way we provide support for the better, but because the situation has changed for the worse, this period makes us offer much more support because the child needs much more support.  “I have the chance to delegate to a lady who helps me in the evening with children, certain routine tasks: bringing them from school, bathing, and supervision during the meal, sometimes making food for children. This allows me to focus on what is essential to me: activities with children and active presence when they are receptive. ”  “I have 3 children. I set out to have a moment of real connection with them each day. It happened to me that after a few days I realized that I had not "seen" my little girl. That I lost something in her development. With the online activity: the boy had an 8-10:30 school program online, then a snack break, then he went to the other room to do his homework. From 11.30 -12.15 the little girl enters the online meetings for kindergarten. After lunch, they were free to play, go out. All the while the baby was in the baby carrier, or on the floor with me playing while we listened to how the homework was done or how the little one was doing online. ” |
| Involvement | Perceived positive changes | “It was very good for us, we even managed to get all the family members involved because otherwise, only two were getting involved”  “What was new in quarantine was that all four of us danced, watched some online concerts that we couldn't get to live, dressed nicely and had dinner like at a restaurant, played more motor games and even acting, we did the lessons together and we walked more in nature and we introduced in the family, especially at the time of the meal, habits from the kindergarten of the little girl who follows the Waldorf program. We also started watching feature films together; we did pop-corn and nesting movies afternoons. ”  “I was very happy every time we invented new games together”  “We interacted during lessons, we interacted during family games on the carpet, we interacted during household activities (washing, cleaning vegetables), we interacted in the evening sleep ritual. ”  “It was ideal for me to have more time to do various experiments and crafts that we couldn't do before."  "I tried to enjoy the time and activities with her as much as I could. We tried to spend more time in nature. "  I'm happy when we have to do activities together. The day is busier, there is a purpose, and it is more colorful. The girl received activities from the kindergarten, the boy received projects from the school. I want them to stay with the memories of the days of the pandemic, not to be the same every day. I feel useful”  "When he has teaching activities, I let him choose his program and I support him when he needs it. I offer him several variants of activities and he can choose what to start with. I don't force him to do what he doesn't like, and when it's mandatory I do it in such a way that he decides. We don't buy unnecessary things anymore, and when he wants something, he thinks about it first” |
|  | Perceived negative changes | "I feel bad when I don't have time for him, I'm sad when he tells me I'm just working, I barely crawl sometimes to play and I accept a maximum of 2 rounds of Monopoly, for example"; "We try to compensate the needs of the child sometimes, but unfortunately the hectic life we ​​have does not allow us otherwise. Eventually, one of us will give up his job and will permanently stay with them”  “Before March, after lunch, after taking the child from kindergarten, I dedicated myself entirely to him, he was exclusively my priority. After March, he often had to wait when he asked me for an activity because I was talking on the phone, or I had urgent situations to solve. ”  “Indeed, jobs consume us all, and the time spent with children for recreational activities is getting shorter and shorter. Limiting travel has further led to a decrease in the various activities performed with the child. |
| Monitoring and control | Perceived positive changes | "I controlled myself very well, I didn't punish him, but I set clear rules: delete all games from the computer, never keep his room closed for hours, never keep the door closed for hours and he would play on weekends or when homework is completed, but not more than half an hour. ”  “Much more independent, both of them. The little one started talking, in the first month of the quarantine, he started riding a bike with pedals, and then quickly without auxiliary wheels. The eldest has learned to be more autonomous in organizing things and finding something to do when he gets bored. ”  "They have become more independent, more responsible when it comes to online classes, homework, and the setting of the table. The little one started classes at 8 o'clock and I had to aid him (being in the 1st grade). The older girls started classes at 9 o'clock, so they prepared their breakfast and served it themselves. "  "The supervision was more rigorous because the volume of individual work was higher than in the period when they were physically going to school. But, I checked to see if they are managed to fulfil deadlines rather than the correctness of their homework, (...) I checked homework randomly or at the express request of the girls”.  "In their free time I imposed 3 rules: no more than 1.5 hours on screens (TV, tablet, and phone), homework is not left to the last minute and in bed at 9.30 pm max." |
|  | Perceived negative changes | “I was more demanding and strict with them. I think he would have liked more lightness, and a slower pace, without worries”  “I ended up playing with him on the phone or the internet half an hour a week so that he wouldn't have to lie or hang out with colleagues in games and forget the notion of time."  "I control him when he's in class (I go into his room to see if he pays attention to class)"  "I imposed several rules, I made a weekly schedule for the time spent in front of the screens but also for extracurricular activities at home, reading, walking. I found that if all this is not put in a clear program (even written and posted on the wall of his room) cannot be done or at least not in a consistent way ”  I became authoritarian. Often, we were no longer just 2 friends, but I was the one who gave the order / and he was the one who has to execute. ”  "I think they are more dependent on us, knowing that we are always with him, he expects us to fulfil more of his cravings."  "He has become more dependent on us in the process of dressing, seeing that we are at home and available, he often asks for help in this regard."  "I think they've become more dependent, because if they used to go to school on their own, now they're constantly asking for help and assistance during class."  “I allowed him to do more things. For example, I extended the time limit for cartoons so that I could complete my work activities. "  "It was a mix between freedom and rules. When I saw the refusal to attend classes, I realized that I could not force him and I left him at his own pace and we did daily activities but chaotically (sometimes in the morning, another time after lunch). And because it was difficult for me to organize my day, I agreed that we needed rules and routines. ”  “I honestly don’t think I managed to keep a balance. I was more authoritative and I imposed clear rules about the school, keeping order in their room. After that, I was sorry and started to apply derogations from the pre-established rules. "  “The only punishment applied was not to watch TV for a week and not use the phone. The punishment with the phone was not effective because the addiction made him hide, but after discussions and an established schedule, he gave stared to give step by step. " |
| Conflict | Perceived positive changes | “Although there were tense moments, I tried to control my emotions much better and if there were conflicts, they quickly ended with a hug, affection, active listening.”  "Most of the time I tried not to react but to listen and understand her. There were times when I failed and we got into an argument. "  "The only problem is the time the child spent on the computer, through negotiation we managed to get over all the difficult moments regarding this matter" |
|  | Perceived negative changes | “Spending most of our time together, me only working from home, I was able to observe him much better and our quarrels were more frequent and were usually due to school activities […] and his desire to stay too long in front of screens and not comply with the established schedule. These reasons have determined strong and frequent tensions between us. "  "I have become a teacher, a role that I did not fulfil successfully. As a result, there were times when situations become tense due to homework."  "The time we spent together was shorter, often leading to frustration on both sides (being present at work throughout the pandemic and with the work overload, not all the time I was very emotionally available after the program" |
| Closeness | Perceived positive changes | “I tried to be more understanding, patient and to listen to their cries, to be close to them and to learn together about their emotions, the way they expressed them and their methods of self-regulation."  "I tried as much as possible to accept the emotions she expressed, to help her recognize them and to regulate them healthily."  "We had more time and more opportunities to connect, to rediscover ourselves, to live in a rhythm that we had in their first years of life."  "We spent more time together and got closer as a family"  "We spent more quality time together, we had more patience with each other, we read more, and we discussed the issues that bothered us." |
|  | Perceived negative changes | "Unfortunately, without much tact and wisdom. I did not understand their needs and failed to help them overcome their fears. "  "I understood that if we don’t impose certain rules (and a certain rhythm), we will not manage to properly get over the quarantine period” So, in short, I brought the army down from the bridge. " |
