# Supplementary material for: Changes in parenting behavior in the time of COVID—19: A mixed method approach
Source: PLoS One. 2024 Apr 19;19(4):e0302125. doi: 10.1371/journal.pone.0302125 (PMC11029621; doi:10.1371/journal.pone.0302125)
Supplement: Attachment — Submitted filename: Rebuttal letter.docx [file pone.0302125.s004.docx]

**Rebuttal letter**

Claudiu Coman

Transilvania University of Brasov

[claudiu.coman@unitbv.ro](mailto:claudiu.coman@unitbv.ro)

Dear Sir/Madam

With this cover letter we submit the revised manuscript, entitled “Changes in Parenting Behavior in the time of COVID – 19: a mixed method approach”, by Luiza Mesesan-Schmitz, Claudiu Coman, Carmen Stanciu, Venera Bucur, Laurentiu Gabriel Tiru, and Maria Cristina Bularca for publication in PLOS ONE.

We revised the manuscript according to the suggestions and recommendation made by the reviewers. We would like to thank the reviewers for taking time to review our paper and for providing such useful suggestions. We also thank the academic editor for reviewing our paper. We tried to comply with all the suggestions and recommendations made by the reviewers, and in this letter, we describe the changes we made to the text according to the recommendations of the reviewers. The changes we made to the text were made while having active the “Track changes” function from Microsoft Word and the text was changed can be best seen while having active the “All markup” option.

**Our response to Reviewer 1:**

We firstly thank the reviewer for taking time to review our manuscript again and provide suggestions in order to improve it. We addressed all the suggestions made by the reviewer. The changes we made to the text can be best seen while having active the “ Track changes” function and the “All markup” option.

**Response to Reviewer 1 comments**

**Reviewer 1 point 1:** Although the topic is interesting, it is not easy to understand what the authors tried to investigate in the introduction section.

**Response 1:** We thank the reviewer for the comment. We would like to mention that in the Introduction section of our initial manuscript, page 9 of the manuscript with “Track changes” and “All markup” option active, we had stated the purpose of our paper and thus we described what we tried to investigate. However, in order to comply with the suggestion of the reviewer, and to state more clearly from the beginning, the focus and the purpose of our paper, we deleted the text that was initially written on page 9 of the manuscript, and we inserted it on page 2 of the revised manuscript, in the Introduction section.

The text we deleted (page 4 of the revised manuscript with “Track changes” and “All markup” option active):

“Taking into account the aspects previously mentioned, the purpose of our paper was to identify the changes in the parenting behavior of Romanian mothers whose children are under the age of 18 in the context of the pandemic, and thus we believed that such an analysis was both relevant and necessary.”

The text we inserted (page 2 of the revised manuscript with “Track changes” and “All markup” option active):

“Taking into account these aspects, the purpose of our paper was to identify the changes in the parenting behavior of Romanian mothers whose children are under the age of 18 in the context of the pandemic. In other words, our paper focused on examining if during the COVID – 19 pandemic, there have been changes in the parenting behavior of Romanian mothers, and on analyzing which aspects of their parenting behavior changed during this period.”

**Reviewer 1 point 2:** Furthermore, in the method section, I am questioning the author's decision to measure parenting skills competencies using an assumption that mothers who have a bachelor's degree could also have certain parenting competencies.

**Response 2:** We are very grateful to the reviewer for raising this point. In order to clarify why we decided to measure parenting skills competencies using the assumption that mothers who have a bachelor's degree could also have certain parenting competencies, in our revised manuscript we provided arguments and we supported our assumption with results of previous studies. In this regard, the previous studies consulted showed that the educational level of parents can influence the way they interact or spend time with their children, and thus can implicitly influence their parenting skills. Hence, on page 14 of the revised manuscript with “Track changes” and “All markup” option active, we inserted an explanation for our decision and we described the results of previous studies, which supported our assumption. We will present further the text we inserted in our revised version of our manuscript.

Text inserted at page 14 of the revised manuscript with “Track changes” and “All markup” option active:

“When considering this assumption, we took into account the results of previous studied which showed the influence of parental education on parents’ behavior in relation with their children. In this regard, the results of a previous study revealed that a mothers’ level of education influences not only the time spent with her children, but also the quality of that time and how she spends time with her children of different ages [29]. Furthermore, previous studies showed that the level of parents’ education, as well as the parents’ income, can have a positive influence on the behavior of the parents at home and on the affective relationship they have with their children [30], and that education, age, or income, can influence the way mothers’ structure the home environment [31], and implicitly their parenting practices.”

Hence, the references we cited in the text were also integrated into the References section of our revised manuscript. The references we added are:

[29] Kalil A, Ryan R, Corey M. Diverging Destinies: Maternal Education and the Developmental Gradient in Time With Children. Demography, 2012; 49(4): 1361–1383. doi:10.1007/s13524-012-0129-5

[30] Davis-Kean PE. The Influence of Parent Education and Family Income on Child Achievement: The Indirect Role of Parental Expectations and the Home Environment. Journal of Family Psychology, 2005; 19(2): 294–304. doi:10.1037/0893-3200.19.2.294

[31] Benasich AA, Brooks-Gunn J. Maternal Attitudes and Knowledge of Child-Rearing: Associations with Family and Child Outcomes. Child Development. 1996; 67(3): 1186–1205. doi:10.1111/j.1467-8624.1996.tb01790.x

*Additional changes: We would like to mention that, because we had to support our assumption with information from previous studies, we cited those studies both in the text and in the References section. Hence, by adding the references mentioned above, the number of the other references changed throughout the revised manuscript. However, all the changes can be seen while having active the “Track changes” function and the “All markup” option.

**Reviewer 1 point 3:** I also didn't get sufficient information about statistical analysis (only spearman correlation?) and the qualitative analysis (independents coder? interrater reliability?)

**Response 3:** We are thankful to the reviewer for the point raised. In order to provide more information regarding the statistical analyses, in section “Materials and methods”, subsection “Measures” we added information about the statistical analyses conducted and information about the qualitative analysis.

At page 15 of the revised manuscript with “Track changes” and “All markup” option active we inserted the following explanation for the quantitative analysis:

“The data were analyzed using IBM SPSS Statistics (version 23). For main measures as Perception of the pandemic impact (PI),Parenting Behavior (PB),Discipline Practices (DP),Parent Involvement in School (PIS) were built index variables as the mean of item’s scale’s scores. The items for each scale related to the concepts are presented in S1_Descriptive statistics of the index's items. Where the scales were used in their initial form, fidelity was tested. Where the scales were adaptations of the original forms, validity was tested. To describe the values of the index variables, percentages, mean, and standard deviation were made. Furthermore, because our research is exploratory, at this stage we tried to identify possible links between variables with the Spearman correlation coefficient between the variables mentioned in the hypotheses, at the sample level, but also on the subsamples given by the categories of the resources variables. Later, they could be tested in a more complex explanatory model. We used this coefficient because it is less sensitive to the type of distribution of the variable scores (in the present case it can also be applied for variables that do not have a normal distribution).”

Furthermore, at page 16 of the revised manuscript with “Track changes” and “All markup” option active we inserted the following explanation for the qualitative analysis:

Moreover, considering the qualitative analysis, we used inter-coder reliability. In this regard, the level of agreement was measured, and two authors who had the role of coders analyzed the qualitative codes established and connected them with the data gathered from the research.”

We would like to mention again that we are thankful to the reviewer for all the points raised and for the time spent on examining our paper!

**Response to reviewer 2**

**Reviewer 2 point:** This manuscript is an excellent start for a series of much needed work looking into parenting styles, shortcomings and acheivements in such specificly unique time of pandemic, which can be widended for further research into parenting methods in thefuture. The limitations were expalined clearly by the authors which allows space and food for thought for future research and studies. I am hoping to see further simialr work by these authors in near future.

**Response to Reviewer 2 point:** We are very grateful to the reviewer for the appreciative words, and we are thankful for the time and effort the reviewer put into assessing our paper!

We are very grateful to the reviewers and the academic editor for all the comments and points raised in order to improve our paper!

Sincerely,

Prof. Dr. Claudiu Coman
